# Soluble Epoxide Hydrolase Inhibition to Face Neuroinflammation in Parkinson’s Disease: A New Therapeutic Strategy

**DOI:** 10.3390/biom10050703

**Published:** 2020-05-01

**Authors:** Mercè Pallàs, Santiago Vázquez, Coral Sanfeliu, Carles Galdeano, Christian Griñán-Ferré

**Affiliations:** 1 Pharmacology Section, Department of Pharmacology, Toxicology, and Therapeutic Chemistry, Faculty of Pharmacy and Food Sciences, Institute of Neuroscience, University of Barcelona (NeuroUB), Av. Joan XXIII 27-31, 08028 Barcelona, Spain; christian.grinan@ub.edu; 2 Laboratori de Química Farmacèutica (Unitat Associada al CSIC), Department de Farmacologia, Toxicologia i Química Terapèutica, Facultat de Farmàcia i Ciències de l’Alimentació, and Institute of Biomedicine (IBUB), Universitat de Barcelona, Av. Joan XXIII, 27-31, 08028 Barcelona, Spain; svazquez@ub.edu; 3 Institut d’Investigacions Biomèdiques de Barcelona (IIBB), CSIC, IDIBAPS and CIBERESP, C/Roselló 161, 08036 Barcelona, Spain; cspfat@iibb.csic.es; 4 Department of Pharmacy and Pharmaceutical Technology and Physical Chemistry, Faculty of Pharmacy and Food Sciences and Institute of Biomedicine (IBUB), University of Barcelona, Av. Joan XXIII, 27-31, 08028 Barcelona, Spain; cgaldeano@ub.edu

**Keywords:** epoxyeicosatrienoic acids, soluble epoxide hydrolase, Parkinson’s disease, neuroinflammation, neurodegeneration

## Abstract

Neuroinflammation is a crucial process associated with the pathogenesis of neurodegenerative diseases, including Parkinson’s disease (PD). Several pieces of evidence suggest an active role of lipid mediators, especially epoxy-fatty acids (EpFAs), in the genesis and control of neuroinflammation; 14,15-epoxyeicosatrienoic acid (14,15-EET) is one of the most commonly studied EpFAs, with anti-inflammatory properties. Soluble epoxide hydrolase (sEH) is implicated in the hydrolysis of 14,15-EET to its corresponding diol, which lacks anti-inflammatory properties. Preventing EET degradation thus increases its concentration in the brain through sEH inhibition, which represents a novel pharmacological approach to foster the reduction of neuroinflammation and by end neurodegeneration. Recently, it has been shown that sEH levels increase in brains of PD patients. Moreover, the pharmacological inhibition of the hydrolase domain of the enzyme or the use of sEH knockout mice reduced the deleterious effect of 1-methyl-4-phenyl-1,2,3,6-tetrahydropyridine (MPTP) administration. This paper overviews the knowledge of sEH and EETs in PD and the importance of blocking its hydrolytic activity, degrading EETs in PD physiopathology. We focus on imperative neuroinflammation participation in the neurodegenerative process in PD and the putative therapeutic role for sEH inhibitors. In this review, we also describe highlights in the general knowledge of the role of sEH in the central nervous system (CNS) and its participation in neurodegeneration. We conclude that sEH is one of the most promising therapeutic strategies for PD and other neurodegenerative diseases with chronic inflammation process, providing new insights into the crucial role of sEH in PD pathophysiology as well as a singular opportunity for drug development.

## 1. Parkinson’s Disease Outline

Due to the increase in population ageing, the incidence of both neurodegenerative diseases and chronic inflammatory conditions are on the rise [1]. Neurodegenerative disorders are now the leading cause of disability in the world, and Parkinson’s disease (PD) is a disabling neurological disease that has the fastest growing index among neurological diseases. PD is a progressive neurodegenerative disease that produces movement disorders. Indeed, it is the most common age-related neurodegenerative motor disease, which implies a gradual loss of motor control that ends up with patients suffering from resting tremors, muscle stiffness, bradykinesia, and postural instability [2]. PD is an insidious onset disease, and the symptoms are subtle at the first stage, being sometimes overlooked, i.e., the lack of tremor during rest does not exclude the diagnosis because it can be absent in 30% of the affected people. Other characteristic signs of PD are hypomimia–hypophonia, dysarthria, sialorrhea, and respiratory difficulties. At the brain level, PD is characterized by the degeneration and loss of neurons in the *substantia nigra pars compacta* (SNc), which causes a selective lack of dopamine (DA), one of the neurotransmitters implicated in regular movements [3]. Lack of DA causes movement control alteration, leading to typical motor symptoms, such as resting tremor or stiffness. Beside for the SNc and the dopaminergic system, other neurotransmission systems can be affected by α-synuclein (α-syn) deposition, including glutamatergic, noradrenergic, serotoninergic, cholinergic, and histaminergic neurons [4]. In fact, the first brain area affected by α-syn deposition appears in the anterior olfactory structures and the dorsal motor nucleus of the vagus nerve, which comprises stage 1 according to Braak theory; afterwards the raphe system and the locus coeruleus can suffer of α-syn deposition (stage 2) [5]. α-Syn reaches the SNc in stage 3, and finally, the hippocampus can also be affected (stage 4). The progression described by Braak shows that noradrenergic and serotoninergic systems are also disturbed in PD. Additionally, specific clinical signs can be explained by noradrenergic dysfunction, which can be significant and anticipate onset of motor symptoms [4]. It is mandatory to keep in mind that the loss of DA in the nigrostriatal pathway is secondary to the axonal degeneration caused by homeostatic disturbances in the SNc [6].

According to the increase in life expectancy of citizens, the number of patients suffering PD duplicated in the last 25 years, and the prevalence will continue growing next years from about 1% to 2% of the world population [7,8,9]. One of the utmost risk factors for developing PD is age [10]. Most commonly, the disease starts between the ages of 50 and 60. Thus, the prevalence increases exponentially from the sixth decade of life. When the PD appears before the age of 50, it is called an early-onset PD. The 95% of PD cases are sporadic; that is, they are not due to a specific genetic alteration [10]. However, it is estimated that between 15% and 25% of people with the disease have a previous familiar history of PD. Additionally, some studies cite as a risk factor continued consumption over the years of well water or exposition to herbicides and pesticides [11].

Although the mechanisms leading to cell death and several of the symptoms of PD are clearly understood, the fundamental question of the etiology of the pathogenesis remains unknown. Furthermore, about the 5% of all cases present symptoms before the age of 60 years, mainly caused by mutations in several genes such as *SNCA*, *LRRK2*, *PARK*, *PINK1,* and *DJ-1* [12,13].

## 2. Therapeutic Strategies for Parkinson’s Disease

To date, there is no curative treatment for PD; therefore, the clinical strategy to treat patients is focused on re-establishing the DA content in the brain to improve the symptoms and quality of life of the patients [13]. The choice of a particular therapy depends on factors such as age, clinical features, and severity of PD and associated disorders. Occasionally, a combination of drug therapy is used for more effective control of symptoms [14]. At present, drugs authorized for PD treatment include L-DOPA, which is a DA precursor, monoaminoxidase inhibitors (MAO)-B (e.g., selegiline, rasagiline), catecol-O-methyltransferase (COMT, e.g., tolcapone, entacapone), as well as DA agonists (e.g., pramipexole, rotigonine) and other anticholinergics or amantadine [15]. Recently, the FDA approved pimavanserin, a serotonin receptor (5-HT2a) inverse agonist, showed decreased frequency and/or severity of hallucinations and delusions without worsening the primary motor symptoms. Nevertheless, all treatments are merely symptomatic but neither modify the progression of the DA associated neuronal loss nor reduce the underlining cellular process that allows the degeneration of the SNc nor the loss of movements control. Pharmacological treatment, although effective, is characterized by undesirable and discouraging motor complications associated with increased occurrence of motor complications such as motor fluctuations (“on–off” phenomena, wearing-off) tremors, or shakes [16]. In parallel, there are practical, although limited, non-pharmacological symptomatic PD treatments mainly effective in motor symptoms including deep brain stimulation of the *subthalamic nucleus* (STN) or the internal part of the *globus pallidus* (GPi) that have complemented these pharmacological approaches [17].

It is noteworthy that we are far from having a completed therapy that delays the progression of the illness, reducing the disabling motor symptoms but also the psychiatric manifestations, like depression, which impair quality of life. Indeed, the efficacy of L-DOPA, the most effective drug, decreases and has unwanted side effects that appear over time. Thus, it is mandatory to face the increase in PD prevalence not only for prevention (by changes in lifestyle such as exercise or diet) [18], reducing risk factors related to the environment (e.g., smoking, pesticides, among others) [19,20,21], or improving the symptomatic treatments, but also developing research programs based on the identification of novel targets and drugs to target them. Likewise, therapeutic strategies that act depending on the stage of the disease will be the optimal scenario to reduce or block its progression. As mentioned, in PD, a progressive but heterogeneous pattern of degeneration occurs, causing a plethora of motor and non-motor symptoms and non-motor alterations [22].

In the last years, a considerable number of drugs that do not have DA activity have emerged and have been tested in clinical phases in an attempt to control the motor fluctuations (on–off phases) related with PD (for a review see [23]) and importantly, to deliver novel disease-modifying therapies. On the one hand, adenosine A_2A_ antagonists (istradefylline, preladenant, and tozadenant (NCT02453386)) have been proposed because adenosine participates in the onset of motor misbalance [24,25,26,27,28]. Istradefylline is another A_2A_ antagonist that has been approved as add-on therapy by the FDA in 2019 (NCT01968031). Thus, caffeine, an A_2A_ antagonist, was described as a putative compound that ameliorates motor and non-motor symptoms of PD (NCT01738178). Noteworthy, the implication of glutamate in the PD has been demonstrated through two classes of receptors: ionotropic receptors (NMDA and his inhibitor amantadine) and metabotropic receptors. Metabotropic glutamate receptors 4 (mGlu4) and 5 (mGluR5) are related to the motor symptoms of PD [24,29]. The use of mavoglurant and diplaglurant (NCT01336088), named as negative allosteric antagonists of mGluR5, and foliglurax (NCT03162874), named as a positive allosteric agonist of mGlu4, have been tested in PD [30,31]. Other non-dopaminergic therapies in development for the symptomatic treatment of PD are serotoninergic drugs, including the 5-HT1a/1b receptor agonist eltropazine (NCT02439125), the 5-HT6/2a receptor antagonist SYN120 (NCT02258152), related with PD dementia, and buspirone (NCT02803749), a 5-HT1a and alpha1-adrenergic receptor agonist. Lastly, several clinical trials based on pharmacological treatments with nicotine (NCT00873392), inosine, a urate precursor (NCT00833690) [32], and isradipine (a calcium channel blocker) started in the last years with different degrees of development and success. For instance, the antihypertensive drug isradipine failed in phase III clinical studies (NCT02168842) but restarted with a new phase III clinical trial design [33]. Creatine monohydrate is an example of repurposing drugs; however, it failed also to improve clinical outcomes (NCT00449865), discouraging the use of creatine monohydrate in patients with PD [34].

The multifactorial pathophysiology of PD challenges the development of novel disease-modifying therapies. Some of these approaches aim to address mutations in crucial genes for PD, including α-synuclein (α-syn), *PARKIN*, and *GBA* [35]. Although most of the studies used α-syn as a biomarker for PD diagnosis and progression [36], α-syn is also a relevant therapeutic target [37] for PD. For instance, α-syn-aggregation modulators (as the use of nilotinib, NCT02281474) or immunization (active or passive) against α-syn is currently studied in clinical trials. An interesting revision on current and researched drugs in PD therapy can be found in Oertel and Schulz (2016) [23]. Several studies also suggest that LRRK2 inhibition is not only useful for mutation carriers’ patients but also for sporadic PD patients [38]. Following this therapeutic strategy, in 2019, Delani Therapeutics started a clinical phase dosing PD patient with DNL151. The *GBA* mutation is closely related to α-synucleinopathy. Thus, GBA inhibitors could also be of interest for patients with sporadic PD. As an example, Venglustat (NCT02906020), a GBA inhibitor, is currently being tested in clinical trials.

Finally, there are other disease-modifying strategies to face PD, but not directly with mutated genes. Several GL1 agonists have entered clinical trials, demonstrating the clinical connection between PD and diabetes mellitus type 2 (DM2) [39]. Exenatide (NCT03456687) and liraglutide (NCT02953665) are GLP1 agonists assayed for the treatment of PD. Finally, the use of coenzyme Q10, a potent antioxidant, failed to demonstrate an improvement in clinical trials [40]. Neither pioglitazone [41,42] nor genomic strategies to increase neurturin (AAV-mediated gene therapy) succeeded in the clinical trials [43,44], since disease-modifying activity was not demonstrated.

Despite all efforts, there is currently no disease-modifying treatment, and hereby it is an unmet medical need that requires an intensification of the research with new strategies focused on disease modification therapies, rather than only symptomatic therapies. As a consequence, this goal stresses the search for new pharmacological targets that open new avenues far from the abovementioned classical approaches to face PD.

## 3. Pathological Hallmarks of PD and the Role of Inflammation

The pathological hallmarks of PD are oxidative stress (OS), mitochondrial dysfunction, abnormal α-syn accumulation, aggregation in Lewy bodies (LB) and other aberrant protein aggregation, and importantly, inflammation [45,46].

Regarding the OS and reactive oxygen species (ROS), both are implicated in dopaminergic neuron damage, which leads to the primary disease characteristic, namely motor dysfunction [45]. Some authors propose that increases of free radicals results from oxidation of cytosolic DA and oxidative metabolism of the neurotransmitter [47]. However, others postulate that the overload of free iron produces the primary cause for damage of SNc and DA neuron death in PD [48]. OS is mainly associated with mitochondrial dysfunction because the mitochondria produces approximately 90% of the cell ROS. Consequently, mitochondrial dysfunction plays an important role in the pathogenesis in PD. Thus, the dynamic mechanisms to maintain the cellular mitochondrial pool and energetic activity such as mitophagy and biogenesis are altered [49].

Deposition of α-syn is another pathological hallmark of PD in the brain, including the striatum. Accumulation of aggregated α-syn is characteristic, and it has been shown in multiple brain regions of PD patients, but its participation in the development of diseases is still poorly understood [50]. Following OS, mitochondrial dysfunction, and α-syn aggregation, neuroinflammation developed and its role in producing neuronal death in PD has been investigated [51,52].

The role of neuroinflammation in PD has been described as an important event involved in pathophysiology. The relevant role of inflammation in PD pathogenesis is evident from the observation that the pro-inflammatory state is a typical hallmark in several age-related diseases, leading to neurodegeneration. Most of the neurodegenerative diseases present inflammatory reactions by activation of the innate immune response as well as the adaptive immune response factors. Concerning brain immune response can be focused on activated astrocytes and microglia, both cell types are able to develop the main neuroinflammatory features, leading to the exacerbation of DA neurons in the SNc [53]. Both cell types release a diverse number of inflammatory mediators, including reactive oxygen and nitrogen species, cytokines, chemokines, prostaglandins (PGs), or complement cascade proteins. Uncontrolled release of those factors can disrupt the blood–brain barrier (BBB), allowing the infiltration of immune cells into the brain. In fact, Braak’s hypothesis on the basic mechanisms for PD onset postulated that a pathogen or environmental toxin provoke local gut inflammation and oxidative stress initiating α-syn deposition in peripheral tissues that can be spread to the CNS, leading to neuronal death. Surviving neurons and microglial cells can then be implicated in a pro-inflammatory mediator release, closing a neuroinflammation cycle processes [5,54].

Interestingly, in PD patients, human leucocyte antigen-DR-positive microglia increases, as well as increases in tumor necrosis factor-alpha (TNF)-α, interleukin-1 beta (IL1-β), interleukin 6 (IL-6), inducible nitric oxide synthase (iNOS), and cyclooxygenase (COX)2 in the striatum and the SNc, being the first evidence of the participation of inflammatory pathways in disease pathogenesis [55]. Of note in models of inflammation such as that given in lipopolysaccharide (LPS) pro-inflammatory lesion, tyrosine hydroxylase (TH), a marker for DA neurons, was reduced in mice [56]. TNF-α was the chief mediator of this neuronal loss because mice lacking TNF-α receptors or TNF-α blockers had delayed PD progression in those mouse models [56,57].

In addition, a recent study has shown that female mice were spared from the same neurodegenerative effects [58]. Microglia in mice pre-primed with LPS in vivo also caused more significant DA neuron loss, as assessed by immunohistochemistry (IHQ), following an insult by an environmental toxin. However, mutant mice that were lacking the gp91 phox subunit of nicotinamide adenine dinucleotide phosphate (NADPH) oxidase (an essential function of the enzyme) showed no difference in IHQ visualization of DA neuron damage. With NADPH oxidase being a key mediator of OS, these findings would support DA neuronal apoptosis via oxidative damage [59]. Finally, other growing evidence supporting the role of neuroinflammation in PD include the implication of inflammatory mediators in the nigrostriatal DA neuron death during the developing of PD [60].

The important involvement of neuroinflammation in PD offers an attractive therapeutic strategy to mitigate the disease. New molecular targets are being proposed that could potentially prevent or delay nigrostriatal death. Those new targets create the opportunity for disease-modifying treatment to what is currently an incurable disease.

## 4. The Role of Soluble Epoxide Hydrolase and Epoxy Fatty Acids in Neuroinflammation

Polyunsaturated fatty acids (PUFAs), namely the ω-3 and ω-6 families, are considered essential components for keeping healthy physiology [61,62,63]. Importantly, these two families of acids have opposite physiological functions; thereby, their ratios have implications in the pathophysiology for some illnesses [64]. The primary actions of PUFAs in the brain include the maintenance of neuronal, glial, and endothelial functions. PUFAs participate in the structure and the maintenance of neuronal, glial, and endothelial cells function in the brain. In mammals, linoleic acid is the ω-6 PUFA precursor of arachidonic acid (ARA) and is provided by dietary vegetables [65]. On the other hand, α-linolenic acid, a ω-3 PUFA, is the precursor of eicosapentaenoic acid (EPA) and docosahexaenoic acid (DHA).

**Figure 1:**
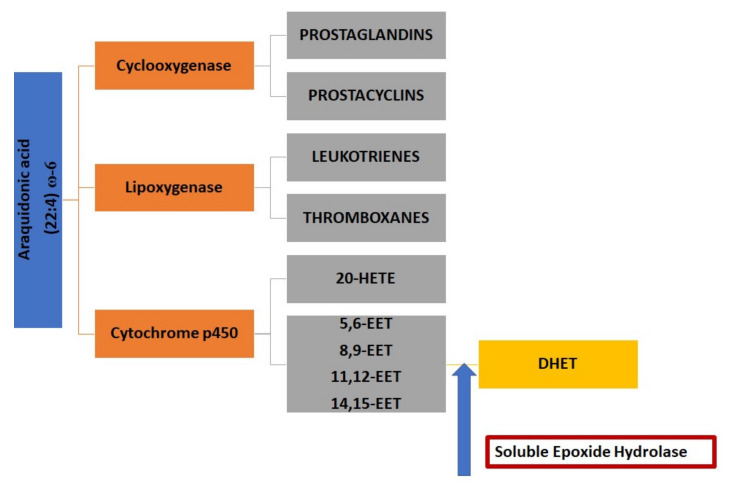
Arachidonic acid (ARA) metabolism tree. Epoxyeicosatrienoics acid (EET) generation and action site of sEH.

ARA, located in the plasma membrane, is released by phospholipids after phospholipase A2 (PLA2) activation. Then, it is metabolized into bioactive derivatives by three main enzymes: cyclooxygenases (COXs), lipoxygenases (LOXs), and cytochrome P450s (CYPs) [63,66,67,68,69,70,71,72]. Prostaglandins (PGs) and thromboxane are produced by COX, leukotrienes, lipoxins, and hydroxyeicosatetraenoic acids (HETEs) by LOX, 20-HETE by CYP hydroxylases and finally epoxy fatty acids (EpFAs), such as epoxyeicosatrienoic acids (EETs), by CYP450 oxidases, including epoxygenases (Figure 1). Concretely, CYP450 epoxygenases produce four EETs regioisomers, i.e., 5,6-, 8,9-, 11,12-, and 14,15-EETs.

EETs and epoxydocosapentaenoic acids (EDPs), as well as some other EpFAs, are regulators of inflammation processes and are particularly important in the brain. The potent anti-inflammatory properties mediated by EpFAs are lost when they are metabolized by soluble epoxide hydrolase (sEH) and the microsomal epoxide hydrolase (mEH) [73,74]. The anti-inflammatory effects of EETs are described in different and diverse animal models. Moreover, EETs also exhibit antioxidant properties, are effective in reducing mitochondrial dysfunction [75] and apoptosis and improve cerebral blood flow [76].

The localization of EETs includes the heart, lungs, kidneys, gastrointestinal tract, and brain. Thus, sEH is expressed in those organs [77]. Regarding the sEH expression in the human brain, the neuronal body, astrocytes, and oligodendrocytes present important levels of sEH [77]. sEH has also been described in meningeal blood vessels and the choroid plexus of the human brain.

sEH (sEH; EC 3.3.2.3) is encoded by a gene, *EPHX2*, localized on chromosome 8, with 45 kb and 19 exons that encode a mature protein of 555 amino acid residues [78,79]. sEH was described first in 1972, and its function is to transform epoxides (EETs) to their corresponding diols [80,81,82,83]. Structurally, human sEH is a bifunctional homodimeric enzyme, showing phosphatase and hydrolase activity. Hydrolase activity is localized in the C-amino terminal domain, whereas the N-terminal domain presents phosphatase activity [84]. The sEH C-terminal domain is in charge of metabolizing endogenous EpFAs substrates [85] and modulating their activity and intracellular fate [86,87].

Of interest, inhibition of sEH improves the beneficial effects of EETs. As an example, in vascular endothelial cells, arteriosclerotic injury causes inflammation by reducing the PPAR-γ activity that is prevented by changes in vascular laminar flow processes. The presence of EETs into vascular endothelial cell preparations leads to an anti-inflammatory effect by increasing PPAR-γ activity [88]. The sEH inhibition (sEHi) through (12-(((tricyclo(3.3.1.1^3,7^)dec-1-ylamino)carbonyl)amino)dodecanoic acid) (AUDA) enhanced PPAR-γ activity in vascular endothelial cells [89].

A few decades ago, it was described that sEHi could be a therapeutic strategy in some age-related diseases through the increased availability of EETs (for revision see [89]). The beneficial effects of sEH inhibitors in the peripheral systems suggest their potential in target neuroinflammation. Different works have linked the anti-inflammatory effect of sEH inhibitors with increases in EET levels that prevent the amplification of the pro-inflammatory cytokines and nitric oxide metabolite levels [89,90]. Given the potent anti-inflammatory effects of the EETs, their endogenous modulation in the brain will be of great interest. Thus, sEHi constitutes a potent new strategy to maintain EES biological activity during disease, being a novel and promising approach for different neurological diseases, including PD.

## 5. Soluble Epoxide Hydrolase in Central Nervous System Disorders

The impressive anti-inflammatory effects of the natural EETs have been described in multiple animal models of several pathologies. They have been tested against diabetes, cardiovascular disease, stroke, traumatic brain injury, PD, epilepsy, cognitive impairment, dementia, depression, and neuropathic pain [89]. Specifically, several reports demonstrated the beneficial effects of sEHi in models of ischemia-induced brain injury [74,91]. Authors suggested that EETs are key regulators of neuronal health and cerebrovascular flow under ischemic conditions, thereby pointing out that the sEHi has a neuroprotective function under those pathological conditions [92,93,94,95]. Likewise, *Ephx2* gene deletion in mice and sEHi by AUDA reduced infarct size after ischemic stroke and had a neuroprotective effect through non-vascular mechanisms [92,96] because EET degradation was prevented. In addition, the beneficial effects of EETs and the sEH inhibitors were also described in animal models of protein misfolding as scrapie [97]. This activity is of particular interest for other neurodegenerative diseases such as PD or AD characterized by an accumulation of aberrant proteins in the brain [97].

Other studies suggest the improvement of cognitive decline induced by vascular alteration, apart from ischemia, for example, the age-related vascular cognitive decline [98]. It is also well documented that EETs produced by perivascular nerves mediated neurogenic vasodilation [95]. Participation of EETs, and therefore sEH activity was reported in rodent models of seizures [99,100]. The beneficial effects reported in those models included both anti-inflammatory properties of EETs or the treatment with sEHi, as AUDA. Interestingly, sEH-KO mice and AUDA-treated mice displayed improved behavior, decreased brain edema, reduced brain tissue damage and apoptosis, and reduced BBB permeability post-traumatic brain injury [101].

It was reported that sEH plays a crucial role in depression [102,103] and in depressive symptoms observed in PD patients and LBD patients [103,104]. Furthermore, there is evidence that confirms the participation of the neuroinflammation, OS, and peripheral and brain lipid metabolism in the pathophysiology of the depression [105,106]. In the case of depression, it has been demonstrated that protein expression of sEH is higher in the prefrontal cortex, striatum, and hippocampus in mice models of depression in comparison with control mice. Furthermore, the most important data are that sEH levels in the parietal cortex from major depressive disorders patients are also higher than in healthy patients. Deletion of the *Ephx2* gene in mice increased the stress resilience after chronic social defeat stress. Worthy of note, sEHi has beneficial effects in animal models of depression [102]. Of note, it was reported that astrocytic EET signaling in the prefrontal cortex is implicated in depressive behaviors [107]. Published data up to now suggests the possibility to establish sEH inhibition as a new approach to treat mood and cognitive symptoms of psychiatric disorders.

Considering that, the importance of neuroinflammation processes in those pathologies has not ruled out thinking to develop experimental strategies to demonstrate the suitability of sEH as a putative target for new treatments in Alzheimer’s disease (AD). The design of new compounds with high efficacy and potency to inhibit sEH, but also with adequate pharmacokinetic properties to enter the CNS, is a challenge that must be faced. Recent studies have been described, such as the use of sEH as a new pharmacological target for neurodegenerative diseases such as PD [108] and AD [109,110]. Recently it has been demonstrated in two mice models of early- and late-onset AD (5XFAD and SAMP8) that treatment with several sEHi reduced neuroinflammation markers, OS and endoplasmic reticulum (ER) stress, after modifying the oxylipin profile, including EETs, the levels of which increased. Interestingly, sEHi impacted on AD hallmarks as amyloid plaques and tau hyperphosphorylation, preventing cognitive decline after oral administration [110]. Additionally, Lee et al. (2019) [111] demonstrated that sEH deletion in an AD mice model (APP/PS1) reduced and delayed the development of cognitive impairment and specific markers of the disease such as β-amyloid deposition and apoE expression.

**Figure 2:**
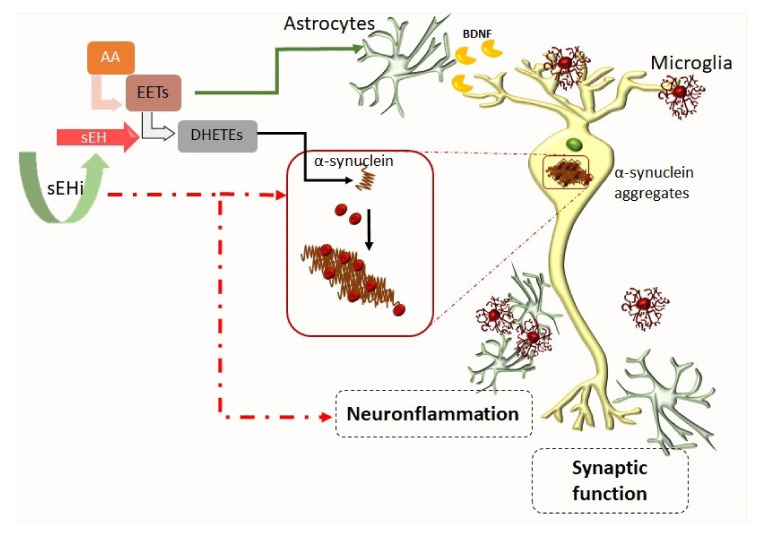
Crosstalk between glial cells, neuroinflammation process, synaptic function, and phosphorylation of α-synuclein favoring aggregation described in PD models [111]. Dotted red line and green line indicate putative regulation role for sEHi.

EET also regulates the neurotrophic role of astrocytes in neurodegenerative disease (Figure 2). To this regard, it was reported that 14,15-EET increase astrocyte-derived brain-derived neurotrophic factor (BDNF) release, increasing the neuroprotective role of astrocytes in ischemic injury [112] and reduced glutamatergic toxicity through astrocytic mGluR5 [113].

Of note, EETs are also related to paracrine signaling, and this means that they could be implicated in the positive action of sEH inhibition [114,115]. sEH activity is associated with the metabolism of EpFAs, altering the levels of EETs and EDPs. Indeed, the more sEH activity increases, the more EETs diminish their paracrine action and can exacerbate the progress or onset of the illness. Thus, it is proposed that the co-treatment with sEHi and ω-PUFAs could be a new approach to consider the different neurological disorders in which a role for sEH and EETs are described.

As mentioned above, sEH has a wide distribution in the organism. Therefore, the increase of EETs in peripheral tissues by systemic sEHi can reduce the systemic inflammatory mediator that could be implicated in beneficial action observed in brain disorders after sEHi treatment [116,117]. The role of EETs increases in peripheral tissues and its impact on CNS disorder development deserves an in-depth knowledge of systemic anti-inflammatory activity mediated by sEHi. However, up to now, an interesting contribution has been anticipated to the prevention and treatment of neurodegenerative disorders with an intense inflammatory process.

## 6. sEH-Phosphatase Activity and Neurodegenerative Diseases

sEH is a bifunctional enzyme, with phosphatase as well as from hydrolase activity [84]. It has been described that increased activity of sEH diminished the cell cholesterol content and lower plasma lipidic levels. However, when hydrolase activity was inhibited, increases in cellular cholesterol and lipid levels were found. The increase in cholesterol content can be linked to the C-terminal domain of sEH, which means a link to the phosphatase catalytic core [118].

Brain cells (neurons and glia) concentrate a quarter of the cholesterol in the body [119]. Isolation of axons by oligodendrocyte myelin sheaths include most of the cholesterol in the brain. Cholesterol is also a key molecule to maintain neuronal morphology and synaptic transmission. Importantly, cholesterol within the brain is synthetized locally because cholesterol does not cross the BBB [120,121]. Consequently, the cholesterol level in the periphery is independent of that in the brain. Nevertheless, there is a constant efflux of this lipid outside the brain, because neuronal cytochrome P450 oxidase Cyp46a1 hydroxylates cholesterol to 24S-hydroxycholesterol (24-OHC), which can be delivered into the circulation, being metabolized by the liver.

As mentioned, it is demonstrated that the sEH-phosphatase domain acts in cholesterol biosynthesis, reducing plasma cholesterol [118,122]. Several neurodegenerative hereditary diseases are associated with disturbances in cholesterol metabolism within the brain, such as Fabry disease, Niemann-Pick Type C disease, Gaucher disease, and GM1 and GM2 gangliosides [123,124,125]. Additionally, the implication of cholesterol metabolism in the ethiopathogenesis of stroke, schizophrenia, depression, and amyotrophic lateral sclerosis, AD or PD, has been described. In the case of AD [126] as an example, it is known that apolipoprotein E allele ε4 increases the risk of AD [127]. Therefore, understanding the effects of the phosphatase domain of sEH on cholesterol levels should open new therapeutic opportunities for disorders where cholesterol can play a role in the onset of the development of the diseases.

## 7. Soluble Epoxide Hydrolase in Parkinson’s Disease

Neuroinflammation is one of the pillars for onset and progression of PD [53]. An early investigation of common polymorphisms of *EPHX1* and *EPHX2*, the genes coding for sEH, in PD patients did not find statistically significant differences in comparison with the healthy control. These first results seemed to discard that sEH expression was important for modifying the risk of developing PD [128]. However, experimental evidence published in the last decade pointed out the implication of sEH in the neuronal processes implicated in the development of PD, opening an alternative for the pharmacological modulation of this enzyme in the therapy of PD. In the same manner as that in depressive disorders, sEH levels in the brain of PD patients were higher than in that of the control [82,96].

MPTP (1-methyl-4-phenyl-1,2,3,6-tetrahydropyridine)-induced parkinsonism is a well-established preclinical in vivo model to study the nigral death (loss in TH-positive cells in the SNc reduced DA and DA metabolites in the striatum) and the motor disabilities characteristics of the disease. However, this model does not develop α-syn aggregates [129,130].

Preclinical testing demonstrated the efficacy of both sEH inhibitors and sEH gene knockout against MPTP-induced Parkinsonism in mice [131]. In this model, sEH deficiency prevented DA (TH-positive) neuronal loss and improved motor activity, measured by the rotarod performance test. Similar results were obtained in a paraquat-induced mouse model of PD, where sEH gene deficiency attenuated TH-positive cell loss [131]. Moreover, the natural anti-inflammatory substrate of sEH, 14,15-EET, protected TH-positive cells and alleviated the rotarod performance deficits of wild-type mice but not sEH-knockout mice, indicating that in the absence of sEH, the endogenous levels of EETs are high enough and then exogenous administration is not useful [131]. Of importance, sEH deficiency neuroprotective effects were lost when 14,15-epoxyeicosa-5(Z)-enoic acid (14,15-EEZE), the 14,15-EET antagonist, was administered to mice, linking the beneficial effects observed directly with this oxylipin (14,15-EET). Furthermore, it has been demonstrated that deletion of the sEH gene protected against MPTP-induced neurotoxicity in the mouse striatum [108,132], while overexpression of sEH in the striatum significantly enhanced MPTP-induced neurotoxicity [108]. Moreover, the levels of the sEH protein in the striatum from MPTP-treated mice were found to be significantly higher than in the control group [108].

Regarding the aberrant aggregation of α-syn in DA neurons, a possible interaction between ω3-acid DHA and α-syn has been postulated, with high levels of DHA found in human brain areas with α-syn [133]. A positive correlation between phosphorylation of α-syn and sEH expression was recently described in PD. Likewise, higher expression of sEH associated with a higher loss in striatal markers such as TH-positive neurons and DA acetyltransferase (DAT) protein levels were found [108]. Authors also suggested a role for sEH in the phosphorylation of α-syn in the mouse striatum because 8,9-epoxy-5Z,11Z,14Z-eicosatrienoic acid (8,9-EpETrE) was able to reduce levels in MPTP-treated mice (Figure 2).

A familial form of PD, named PARK2, is caused by a mutation in the *PARKIN* gene [134]. Interestingly, human induced pluripotent stem cell (iPSC)-derived neurons from PARK2 patients showed a higher *EPHX2* gene expression and apoptotic death compared with control neurons [108]. The sEH inhibitor 1-(1-propionylpiperidin-4-yl)-3-(4-(trifluoromethoxy)phenyl)urea (TPPU) was able to reduce apoptosis in the human PARK2 iPSC-derived neurons, reinforcing the hypothesis that increased sEH expression in the striatum may be implicated in the development of PD. Moreover, pharmacological inhibition by the sEH inhibitor TPPU or sEH deficiency protected against MPTP-induced OS in the mice striatum. MPTP induces several stressful signs in neurons, as ER stress, and inhibits beneficial pathways such as the Akt pathway. MPTP reduces DA and DA metabolite levels in the striatum of treated wild type (WT) mice, but these effects were prevented by TPPU and were undetectable in sEH deficient mice [108]. Likewise, sEH deletion reduced MPTP-induced ER stress in the striatum, and pre-treatment with TPPU had similar beneficial effects [108]. Moreover, Qin and collaborators [131] determined that the sEH deficiency does not allow the inactivation of the Akt pathway induced by MPP^+^ in neuronal cultures observed in WT cultures. They hypothesized that sEH deficiency increases 14,15-EET in MPTP-treated mice, which in turn activates the Akt neuroprotective pathway, preventing TH-positive neurons loss and behavioral dysfunction.

There is a growing interest in the implication of systemic inflammation on the onset and progression of neurodegenerative diseases, including PD [135]. Recent studies also highlight the role of the immune system in the pathogenesis of PD. Environment and aging impact the immune system, and there is strong evidence that the immune system is important in the onset and early phases of PD. Importantly, the inflammatory process is more than a consequence of disease, contributing inexorably to the progression of striatum degeneration [136,137]. GWAS studies demonstrated that several genes related to higher susceptibility to develop PD are related to immune functions [138,139]. Furthermore, genes mutated in familial or sporadic PD (such as *PARK* or *LRRK2*, *DJ-1*) are present in microglia and astrocytes [140]. Finally, several polymorphisms in genes related to pro-inflammatory factors (such as interleukins or human leukocyte antigen (HLA) complexes) have been demonstrated to increase PD susceptibilities [141].

**Figure 3:**
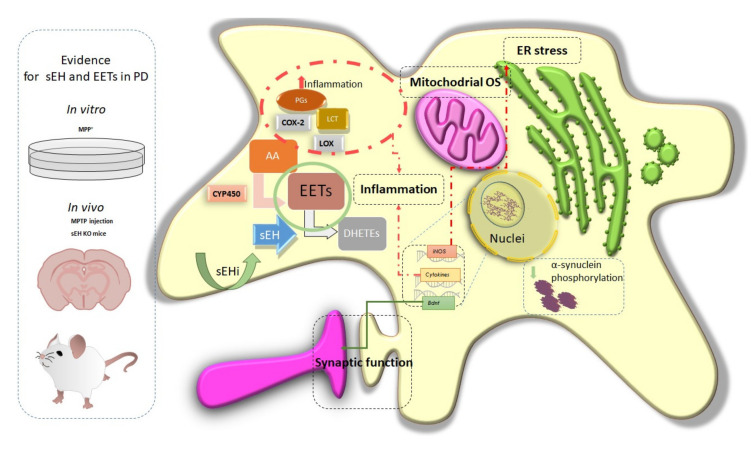
Representative scheme of sEH implication in PD and hot spots of pharmacological activity for sEH modulation. Dotted red line and green line indicated putative regulation role for sEHi.

To sum up, the previously published results suggested that sEH inhibitors or deletion might be able to protect DA neurons from death. Thus, giving the role that neuroinflammation, OS, and mitochondrial dysfunction play in PD and the efficacy of inhibiting the activity of sEH in preclinical models, sEH is a putative new pharmacological target for the treatment of PD and other α-syn-related pathologies such as Lewis body disease (LBD) [142] (Figure 2 and Figure 3).

Finally, as mentioned, there are neurodegenerative diseases for which a contribution of impaired cholesterol metabolism to the disease has been proposed. For PD, several reports also indicated lysosomal system dysfunction associated with the pathogenesis of PD [143]. Of note, alteration in cholesterol metabolism has been found in PD patient-derived fibroblasts [144] and in the serum lipid profile of PD patients [145,146]. As mentioned above, an inverse correlation among sEH hydrolase and phosphatase activity on cholesterol synthesis has been described [118]. Therefore, it should not be discounted that the use of sEHi for PD can have two-fold effects to treat disease, namely on neuroinflammation, by increasing EETs, and in improving cholesterol trafficking in brain cell membranes.

## 8. Therapeutic Use of sEH Modulation in Parkinson’s Disease

sEH could likely represent a promising therapeutic target for neurological disorders such as depression, PD, LBD, and AD [108,110,111,147]. In the recent past, several reports demonstrated the usefulness of small molecules for inhibiting sEH activity, which was effective against several illness conditions such as cancer, hypertension, heart diseases (ischemia, cardiac, and renocardiac failures), obesity, and diabetic neuropathy [148,149,150,151,152,153,154,155,156]. Compounds with building blocks containing ureas, thioamides, thioureas, acyl hydrazones, or carbamates, among others, presented a potent and specific effect inhibiting sEH [157,158]. Such natural compounds as honokiol [153,159] or 1,3-bis (4-methoxybenzyl) urea (MMU), an abundant component of *Pentadiplandra brazzeana (P. brazzeana)* was also able to inhibit human sEH at nanomolar concentrations [159]. Interestingly, traditional medicinal use of the root of *P. brazzeana* in patients with psychiatric and neurological disorders was reported [158].

It was demonstrated that EET release from astrocytes and DA neurons in co-culture increased cellular defense and viability in the face of OS [160], suggesting that sEH inhibition can offer a neuroprotectant role to DA neurons in PD. Indeed, the sEH inhibitor AUDA increases EET levels, enhancing the neuronal viability, suggesting a neuroprotectant role of EET in the brain after an oxidative environment in brain, as occurs in PD or other neurodegenerative diseases. Besides, use of EETs in primary sensory and cortical neurons culture induces an increase in the axonal outgrowth [161]. This effect anticipates a possible action of sEH inhibitors as a nerve regenerating therapy [161].

It was also demonstrated that the promitogenic effect of directly injected EETs induced an endothelial proliferation, being implicated in angiogenesis in cerebral vasculature [44,76,95,155] Concretely, in a co-culture of astrocytes and cerebral microvascular endothelial cells, EETs were able to increase the endothelium tube [162]. On the other hand, the beneficial role in the CNS of this promitogenic activity induced by EETs was not completely demonstrated. On the other hand, reports indicated that sEHi in specific areas of the brain, such as the brainstem, increased the blood pressure and induced tachycardia in hypertensive animals but not in normotensive ones [163]. Likewise, sEHi exerted a vasodilatory action [149].

Lakkappa and coworkers demonstrated that the sEH inhibitor PTUPB provided neuroprotection in a *Drosophila melanogaster* model of PD [142]. Moreover, AUDA pre-treatment reduced TH-positive neuronal loss induced by MPTP, but this beneficial effect was not observed after AUDA post-treatment [131]. This preventive but not reversing effect of MPTP neurotoxicity does not discourage the use of sEH inhibitors as a therapeutic strategy but indicates that the putative treatment should be implemented at the early stage of PD. Recently, Ren et al. [108] demonstrated that TPPU, in an oral administration, prevented the loss of DAT, TH, and ER stress in the SNc and striatum after MPTP treatment in mice, and prevented the apoptosis of *PARK2* iPSC-derived neurons.

In conclusion, the use of sEHi in a PD in vivo model strengthened the cell culture clues about the possible use of those compounds in the therapy of PD, both at an earlier stage and as an add-on therapy. Table 1 lists the published works on sEH, sEHi, and PD, and Table 2 summarizes sEHi tested in PD models and its chemical structures. Those works were the basis for this review. Several authors also proposed sEH inhibitors as a prophylactic therapy to prevent the progression of the disease. The antioxidant and, importantly, the anti-inflammatory role of sEHi are the keys to the beneficial effects of sEHi in PD. It is noteworthy that ω3-PUFAs present neuroprotective effects in the face of several neurological disorders. It was reported that the use of an EPA-enriched diet in mice treated with MPTP, a model of PD, diminished the hypokinesia and improved memory induced by the dopaminergic toxic [164]. In humans, the dietary supplementation with PUFAs diminished the risk of developing PD [165,166]. The neuroprotectant role of dietary PUFAs can be associated with the fact that those compounds must be incorporated through food intake. Linolenic acid is the predominant ω-3 PUFA from the diet, and a precursor of EPA, DHA, and AA. A PUFA-enriched diet influences the increase of EETs, which in turn exert their beneficial effects in CNS.

## Figures and Tables

**Table 1:** Publications crosslinking sEH and PD or other central nervous system (CNS) disorders.

	Outline	Biological Substrate	Reference
The protective effect of astrocyte-derived 14,15-epoxyeicosatrienoic acid on hydrogen peroxide-induced cell injury in astrocyte-DA neuronal cell line co-culture.	Implication of sEH in PD pathology	Astrocyte-DA neuronal cell line co-culture	[160]
Soluble epoxide hydrolase deficiency or inhibition attenuates MPTP-induced parkinsonism	Implication of sEH in PD pathology	Mice	[131]
Soluble epoxide hydrolase plays a key role in the pathogenesis of Parkinson’s disease.	Implication of sEH in PD pathology	Human, mice	[108]
Role of epoxy-fatty acids and epoxide hydrolases in the pathology of neuro-inflammation.	Role of EETs in neuroinflammation	Review	[167]
Evaluation of antiparkinson activity of PTUPB by measuring dopamine and its metabolites in *Drosophila melanogaster*: LC–MS/MS method development.	Therapeutic profile for sEHi in PD	*Drosophila melanogaster*	[168]
Humble beginnings with big goals: Small molecule soluble epoxide hydrolase inhibitors for treating CNS disorders.	Therapeutic profile for sEHi in CNS disorders	Review	[89]
Soluble epoxide hydrolase inhibitor, APAU, protects DA neurons against rotenone induced neurotoxicity: Implications for Parkinson’s disease.	Therapeutic profile for sEHi in PD	DA cell culture	[169]
Role of soluble epoxide hydrolase in metabolism of PUFAs in psychiatric and neurological disorders.	Therapeutic profile for sEH	Review	[158]
Cytochrome P450 derived epoxidized fatty acids as a therapeutic tool against neuroinflammatory diseases.	Role of EETs in PD	Review	[170]

**Table 2:** Compounds tested in Parkinson’s disease models.

Compound	Chemical Structure	Reference
Honokiol (5,3′-diallyl-2,4′-dihydroxybiphenyl)	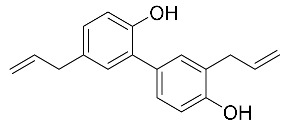	[153,160]
12-(3-(adamantan-1-yl)ureido)dodecanoic acid (AUDA)	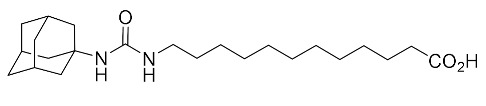	[92,96]
1,3-bis (4-methoxybenzyl)urea (MMU)	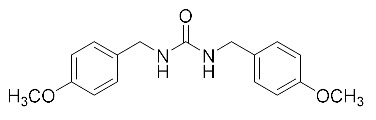	[159]
N-(1-acetylpiperidin-4-yl)-N-(adamant-1-yl)urea (APAU)	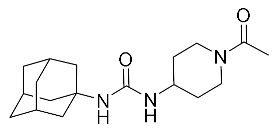	[170]
(4-(5-phenyl-3-{3-[3-(4-trifluoromethyl-phenyl)-ureido]-propyl}-pyrazol-1-yl)-benzenesulfonamide) (PTUPB)	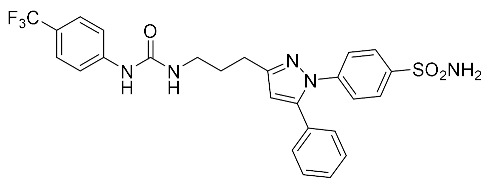	[143]
1-((1-Propionylpiperidin-4-yl)-3-(4-(trifluoromethoxy) phenyl)) urea (TPPU)	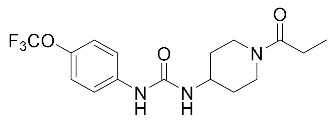	[109]
